# Imputation-free reconstructions of three-dimensional chromosome architectures in human diploid single-cells using allele-specified contacts

**DOI:** 10.1038/s41598-022-15038-4

**Published:** 2022-07-11

**Authors:** Yoshito Hirata, Arisa H. Oda, Chie Motono, Masanori Shiro, Kunihiro Ohta

**Affiliations:** 1 Faculty of Engineering, Information and Systems, University of Tsukuba, 1-1-1 Tennodai, Tsukuba, Ibaraki 305-8573 Japan; 2 Department of Life Sciences, Graduate School of Arts and Sciences, The University of Tokyo, Meguro-ku, Tokyo, 153-8902 Japan; 3 Cellular and Molecular Biotechnology Research Institute, National Institute of Advanced Industrial Science and Technology, Koto-ku, Tokyo, 135-0064 Japan; 4 Computational Bio Big-Data Open Innovation Laboratory (CBBD-OIL), National Institute of Advanced Industrial Science and Technology (AIST), Waseda University, 3-4-1 Okubo, Shinjuku-ku, Tokyo, 169-0072 Japan; 5 Mathematical Neuroscience Research Group, Human Informatics and Interaction Research Institute, National Institute of Advanced Industrial Science and Technology (AIST), Tsukuba, Ibaraki 305-8568 Japan; 6 Research Center for Complex Systems Biology, Universal Biology Institute, 3-8-1 Komaba, Meguro-ku, Tokyo, 153-8902 Japan

**Keywords:** Computational biology and bioinformatics, Molecular modelling

## Abstract

Single-cell Hi-C analysis of diploid human cells is difficult because of the lack of dense chromosome contact information and the presence of homologous chromosomes with very similar nucleotide sequences. Thus here, we propose a new algorithm to reconstruct the three-dimensional (3D) chromosomal architectures from the Hi-C dataset of single diploid human cells using allele-specific single-nucleotide variations (SNVs). We modified our recurrence plot-based algorithm, which is suitable for the estimation of the 3D chromosome structure from sparse Hi-C datasets, by newly incorporating a function of discriminating SNVs specific to each homologous chromosome. Here, we eventually regard a contact map as a recurrence plot. Importantly, the proposed method does not require any imputation for ambiguous segment information, but could efficiently reconstruct 3D chromosomal structures in single human diploid cells at a 1-Mb resolution. Datasets of segments without allele-specific SNVs, which were considered to be of little value, can also be used to validate the estimated chromosome structure. Introducing an additional mathematical measure called a refinement further improved the resolution to 40-kb or 100-kb. The reconstruction data supported the notion that human chromosomes form chromosomal territories and take fractal structures where the dimension for the underlying chromosome structure is a non-integer value.

## Introduction

The three-dimensional (3D) chromosomal structure plays important roles in various biological processes such as DNA replication and gene regulation. There are two major methods to investigate the 3D chromosomal structures: (1) microscopy-based fluorescent in situ hybridization (see Ref.^1^ for the review) and (2) chromosome conformation capture techniques combined with deep sequencing and a computational reconstruction (Hi-C)^2^. The Hi-C method has been applied to individual human cells^3–5^. Although several methods have been developed to reconstruct the 3D chromosomal structure in human haploid cells^6–8^, the available methods for diploid cells are mostly for ensemble Hi-C data^9,10^ (see also reviews^11,12^).

Among them, the method by Carstens et al.^7^ has been used for a single diploid cell. They combined ambiguous distance constraints with the inverse sixth powers of distances to realize the “OR” operation, or the circumstance where chromosome segments of a paternal or maternal allele satisfy some distance constraints. They claim that bias can be avoided in assigning alleles for each contact. However, their results are validated only from the viewpoint of consistency with preexisting results.

A previous report demonstrated an experimental method and its accompanying computational method called imputation, which was proposed to overcome the sparseness of the Hi-C dataset of single diploid cells^13^. They distinguished two alleles on each homolog by differently labeled single nucleotide variations (SNVs). Then they imputed unlabeled alleles using the information of neighbors by assuming that different alleles typically contact different chromosomal segments. Specifically, they made the following assumptions: (i) two alleles are not close to each other, and (ii) alleles do not have similar shapes^13^. Lastly, they used the imputed allele labels to reconstruct the 3D chromosomal structure with simulated annealing. Since the frequency of SNVs should be insufficient to mark all the sequence segments read from different alleles, only a few percent of segments contain enough information to identify the derived allele. The rest contain only ambiguous information (Supplementary Table 1). Imputation tries to employ this ambiguous segment information to obtain a high-resolution reconstruction. This Hi-C reconstruction algorithm for a single diploid cell is quite powerful, but such imputations may contain the above-mentioned assertions that cannot be verified directly.

Here, we propose an alternative imputation-free computational method to reconstruct the 3D structure from Hi-C data for a single diploid cell. This method is an extension of our previous recurrence plot-based reconstruction method^8^. The key feature of our method is that only consecutive chromosome segments are assumed to be neighboring. To estimate a 3D chromosome structure, we use only the parts of pairs where both alleles on homologs contain SNVs. The remaining pairs of chromosome segments, which have at least one allele without SNVs, were used for the self-validation of the estimated 3D chromosomal structures. Furthermore, we refine the initial reconstruction of 1-Mb resolution to that of 40-kb or 100-kb resolution (see Table 1 for the overall summary of the current work). Finally, we discuss the validity for the reconstructed 3D structure by checking the similarity and difference between 3D structures for allele pairs.

**Table 1:** Comparison of our previous method (Hirata et al., Sci. Rep. 2016) with the current work.

	Discrimination of homologous chromosomes	Refinement process
Hirata et al. (2016)	No	No
The current work	Yes	Yes

## Method

### Recurrence plot-based reconstructions

We use the similarity between single-cell Hi-C data and a recurrence plot^14,15^ to reconstruct the 3D structure for corresponding chromosomes^8^. We apply the same strategy to reproduce the 3D structure from single-cell Hi-C data. However, the present study has two differences compared with the method of Ref.^8^. First, only segment pairs containing SNVs are used to calculate local distances between segment pairs. Second, this study initially obtains a coarse reconstruction using the method of Ref.^8^, which is subsequently refined by employing the idea of time series forecasting^16^.

### Discrimination of homologous chromosomes using data of paired segments with allele-specific SNVs

This section describes the differentiation of two homologous chromosomes for a single diploid cell in the proposed method.

**Figure 1:**
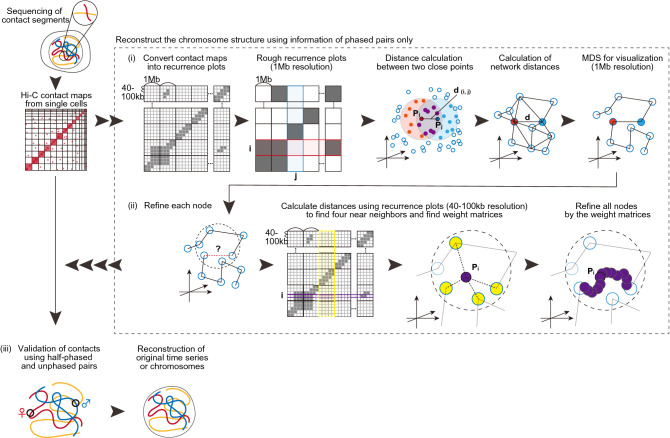
Graphic summary of the reconstruction of the 3D structure for chromosomes given single-cell Hi-C data using the recurrence plot-based method.

For an ensemble of diploid cells, a Bayesian technique can be used to differentiate maternal alleles from paternal alleles^7^. For the single diploid cell data presented in Tan et al.^13^, we experimentally used the SNVs to differentiate one allele from the other as a potential genetic marker from the sequencing information. Thus, we focus on pairs of chromosome segments, which are spatially close enough to be detected by the Hi-C experiment and contain SNVs (Table 2). Only these pairs of chromosome segments (phased pairs) are used. We used phased pairs of contacts identified in the accompanying datasets by Tan et al.^13^. Then, we apply the algorithm of Ref.^8^ to reproduce the 3D structure for the chromosomes (Fig. 1).

**Table 2:** How we treat the single diploid Hi-C data.

	Second segment with SNVs	Second segment without SNVs
First segment with SNVs	3D structure reconstruction	Cross-validation
First segment without SNVs	Cross-validation	Cross-validation

Thus, we can use pairs of spatially close chromosome segments where at least one of them may not contain the sequence of SNVs to verify the reconstructed 3D structure (Supplementary Fig. 1). Namely, if two chromosome segments are detected as a contact in the single cell Hi-C data (which we hereafter call a neighbor), one of the chromosome segments can be identified by its original allele while the origin of the other allele is unknown (half-phased pairs). For half-phased pairs, the distance of the identified segment to the corresponding location of one of two allele segments should be close. If two chromosome segments are detected as neighbors, and neither contains SNVs (unphased pairs), it is impossible to tell which alleles they are from, but the closest distance among four possible pairs between the corresponding allele segments should be close. We will validate this tendency in “Radial distance for each allele” section (see also Table 2). Therefore, the novelty of the current work is (i) it applies the method of Ref.^8^ only on phased pairs of single diploid cell Hi-C data, (ii) the reconstruction is refined, and (iii) half-phased and unphased pairs validate the reconstruction (see Table 2).

### Summary of the proposed method

The computational procedure of our reconstruction can be summarized as follows. First, we declare that consecutive chromosome segments on the same allele are neighbors^8,17^ and neighbors are defined by phased pairs. Second, we construct a network from the Hi-C map^8,18,19^, where each node corresponds to a chromosome segment and each contact corresponds to an edge. Then, we assign a local distance to each edge. The local distance between two chromosome segments can be determined by the ratio of the unshared neighbors against the union of the neighbors at their corresponding rows^8,18^. Third, we obtain the shortest distances between every pair of nodes^8,18^. The shortest distances can be regarded as the global distances^20^. Fourth, we convert the global distances into point arrangements in 3D space while preserving the distances via multidimensional scaling^21^. These point arrangements correspond to our coarse 3D reconstruction at a 1-Mb resolution. Lastly, the four closest neighbors are found for each point at a 40-kb or 100-kb resolution based on the similarity of the connected nodes. Then the weighted averages^16^ of the neighbors’ coarse 3D reconstruction at a 1-Mb resolution are used for a finer reconstruction at a 40-kb or 100-kb resolution. If the last step is removed, then the proposed method coincides with our previous method^8^. In addition, if all the local distances are approximated by 1 at the second step, our method agrees with the single-cell Hi-C implementation of Ref.^22^, while our local distance calculations can be considered as weighted by Jaccard coefficient. It should be noted that Ref.^6^ defined local distances as a constant for single-cell Hi-C data as well as the paper employed a manifold based learning technique instead of the shortest distance approach used here.

Figure 1 shows a graphic summary of how we reconstructed the 3D structure of the chromosomes from single-cell Hi-C data. In addition, the Supplementary Material contains the mathematical details for the above calculations.

### Validation using protein/polymer models

In this section, we verify our proposed method by comparing the relatively close method of Ref.^22^ using some biopolymers with known 3D structures. First, we checked the validity of the proposed method without refinement on protein data as smaller linear biopolymers because (i) both proteins and chromosomes can be described as contact maps, where a set of contact pairs are visualized in a two-dimensional space; (ii) protein structures have been investigated more deeply than chromosome structures to date. The results are presented in Supplementary Fig. 2. There, Panel (a) shows that a contact map is well preserved after reconstructing the protein 3D structures. Panel (b) shows that our reconstructed protein 3D structures are more similar to their truths than the cases where all the local distances are set to 1. Therefore, the proposed method seems to work finely before its refinement process. Please see Supplementary Material for detail.

As a second test, we examined the proposed algorithm with the last step of the refinement process. Here, we used a polymer simulation of chromosomes at a 1-Mb resolution by the previous method^23^. We varied two parameters. The first was the threshold for defining the closeness, or the recurrence rate, which shows the ratio of intersections where contacts exist. The second was the number of points that kept contact information used for our reconstruction. During the coarse reconstruction, every fifth point was used as a reconstructed point. The reproduced contact map has high accuracy even if the underlying 3D structure is inferred only from a portion of contacts and then the contact map is reproduced (Supplementary Figs. 3 (a) and 3(b)). Supplementary Figs. 3(c) and 3(d) show that the values of the 3D correlation coefficients for the proposed method are 0.9 or higher, indicating that the original shape is mostly preserved after the reconstruction even if a large portion of points is discarded (Supplementary Fig. 3(d)).

A further examination of the results showed that the 3D correlation coefficient with the original shape tends to be systematically higher for our reconstructions than the simple application of the previous report^22^ followed by refinement (Supplementary Fig. 3(c) and 3(d)). This result demonstrates that our proposed framework can reconstruct finer detailed structures more effectively. This may be because the ratio of points used to estimate the local distances in the proposed method is a robust quantity under uniform sparsity.

## Results

We analyzed the datasets of Ref.^13^. There are two types of cells: GM cells (GM12878), which are a female human lymphoblastoid cell line, and peripheral blood mononuclear (PBMC) cells. The datasets were downloaded from www.ncbi.nlm.nih.gov/geo/query/acc.cgi?acc=GSE117876 with the GEO Series accession number GSE117876. We used their “clean” datasets for our reconstructions. There, all the contact information, as well as the phase information used here, were provided, and thus we did not conduct any sequencing analysis. Below, we show the results of 15 GM cells and all 18 PBMC cells. One GM cell (GM cell 8) had a missing dataset. Additionally, our reconstruction was not completed for another (GM cell 10), which may be because the chromosomes are separated into two pieces or more.

### Fractal globule and chromosome territories

**Figure 2:**
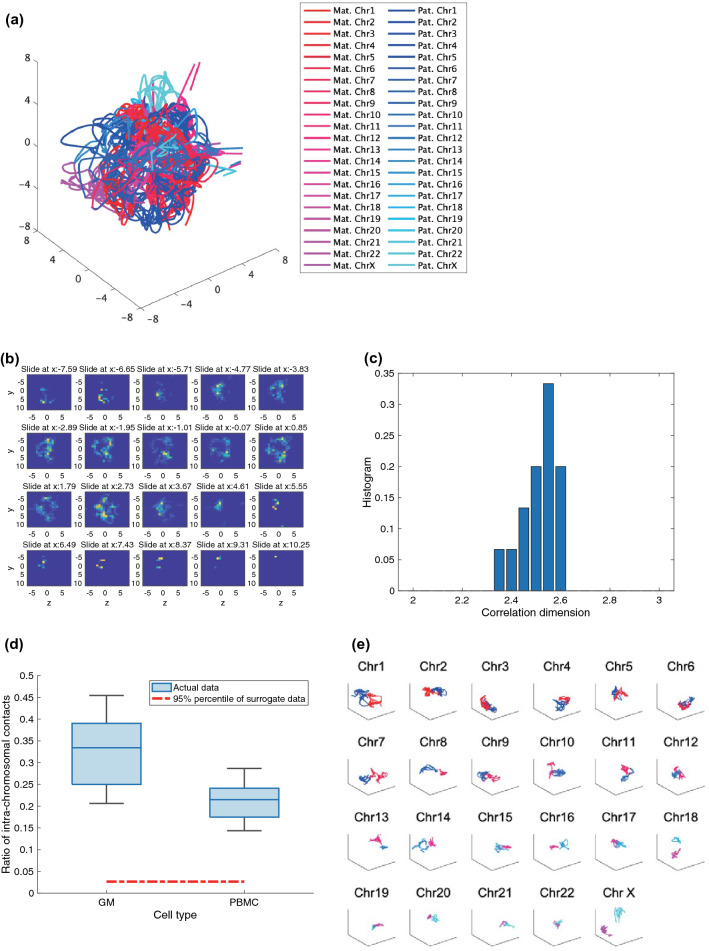
3D structure for the reconstructed chromosomes from single-cell Hi-C data. (**a**) Our 40-kb reconstruction for GM cell 2. (**b**) Density plot for GM cell 2 at a 40-kb resolution. There is a hole in the center. (**c**) Correlation dimensions for GM cells at a 100-kb resolution. The correlation dimensions are not integer, implying the fractalness for the underlying chromosome structure. (**d**) Ratio of intra-chromosomal contacts for GM cells as well as PBMC cells at a 100-kb resolution compared with 20 randomly shuffled reconstructions. Panel (**d**) means that there are chromosomal territories for each of GM and PBMC cells. Panel (**e**) shows the three-dimensional structures with more detail for GM cell 2. Each sub-panel shows a chromosome where reddish and bluish colors, which are the same as Panel (**a**), indicate maternal and paternal alleles, respectively.

Figure 2a shows a typical example of our reconstruction. The set of chromosomes forms a sphere. The center typically has a hole (Fig. 2b). We normalized the radial distance for the hole by the mean radial distance for the reconstructed points. Then we compared the value obtained for human lymphocytes from their nucleolar area^24^ and nucleus volume^25^ (Supplementary Fig. 4). GM cells are lymphoblastoid cells, which originate from lymphocytes. The values obtained for our reconstructions are close to the estimated values for human lymphocytes. Thus, we presume that this hole corresponds to the nucleolus. In addition, the values obtained for our reconstructions are more consistent with the estimated values than those for the previous reconstructions^13^.

Then we estimated the correlation dimensions using the method descrived previously^26^. In short, the correlation dimension quantifies the scaling factor of how fast the number of points within a certain distance grows when we increase the distance within which two points are regarded as close. The logarithms for the accumulated proportions of the spatial distances are linearly scaled with the logarithms for the spatial distances (Supplementary Fig. 5). Moreover, the values of the correlation dimensions are between 2 and 3 (Fig. 2c). However, the dimension for the chromosomal structure as judged by the distribution is likely to be considerably below 3 (average 2.5126, s.d. = 0.0745), implying a fractal chromosome structure. The values obtained were similar to the ones in the previous study^27^.

It has been argued that the fractal nature of the chromosome structure leads to “chromosomal territories”^28^. Thus, the evaluated ratio of intra-chromosomal contacts suggests chromosomal territories rather than randomly shuffled points of chromosomes (Fig. 2d). Therefore, our observations imply the existence of chromosomal territories. Plotting the two alleles of each chromosome separately shows that alleles are clustered (Fig. 2e). Thus, our findings are similar to those in the previous report^13^.

### Reconstruction consistency

**Figure 3:**
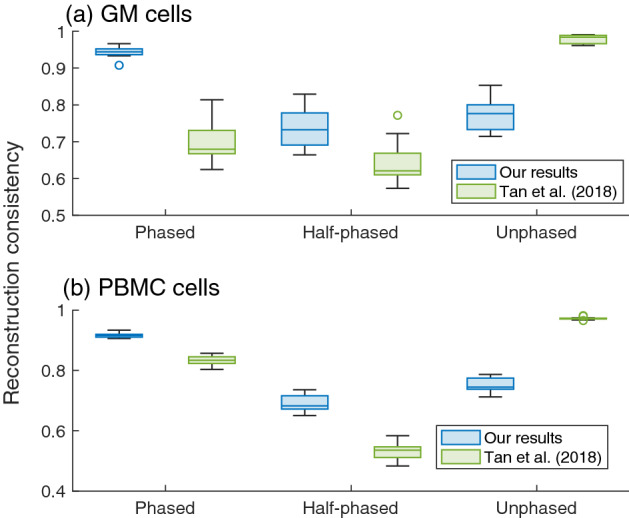
Self-checking results of the reconstructed 3D structure at a 40-kb resolution. Ratios where two close segments in a chromosomal contact for a single Hi-C are within the detection limit distance of 22/27 in the proposed method (see Supplementary Material for the derivation of the number “22/27”), depending on three conditions: both segments with SNVs (phased), only one of two segments with SNVs (half-phased), and both segments without SNVs (unphased). For obtaining the ratios, we used the number of all the corresponding contacts in each single diploid cell Hi-C data as the denominator for each cell. For the results of Ref.^13^, the detection limit distance is obtained by multiplying 22/27 and the ratio of the estimated mean distance for the reconstructions of Ref.^13^ against that of our results. Panel (**a**) GM cells and (**b**) PBMC cells, where our results are on the left and those of Tan et al.^13^ are on the right.

Although our observations support a fractal globule forming chromosomal territories, there are differences between our reconstructions and those by Ref.^13^. Our reconstructions have a higher consistency for phased pairs and half-phased pairs than those by the report by Tan et al.^13^ (Fig. 3). Additionally, their analysis^13^ marked a higher value of reconstruction consistency for unphased pairs (Fig. 3). This artificially high value is due to their method, where their imputation process, or assigning alleles with voting by neighbors, forced to create clusters of the same alleles, while these clusters could be spurious. In fact, if each chromosome is evaluated, only our reconstructions in the sex chromosomes in PBMC cells are similar to those in the report by Tan et al.^13^ (Supplementary Fig. 6(b)). Our reconstructions for other chromosomes differ (Supplementary Figs. 6(a) and 6(b)). In the sex chromosomes, the two reconstructions look similar because the report by Tan et al.^13^ did not have to impute alleles. Hence, our reconstructions are more consistent with a given single diploid cell Hi-C dataset, as the imputations in their analysis may generate some bias.

Furthermore, two alleles on chromosomes 9, 13, 14, 15, 21, and 22 have similar shapes (Supplementary Fig. 6(c)). For these chromosomes, the cell-to-cell variability seems negligible (Supplementary Fig. 6(d)). Moreover, the two X chromosomes in female-derived GM cells look different in the top panel of Supplementary Fig. 6(c). This may be due to the inactivation of one of the two X chromosomes^29^.

### Radial distance for each allele

**Figure 4:**
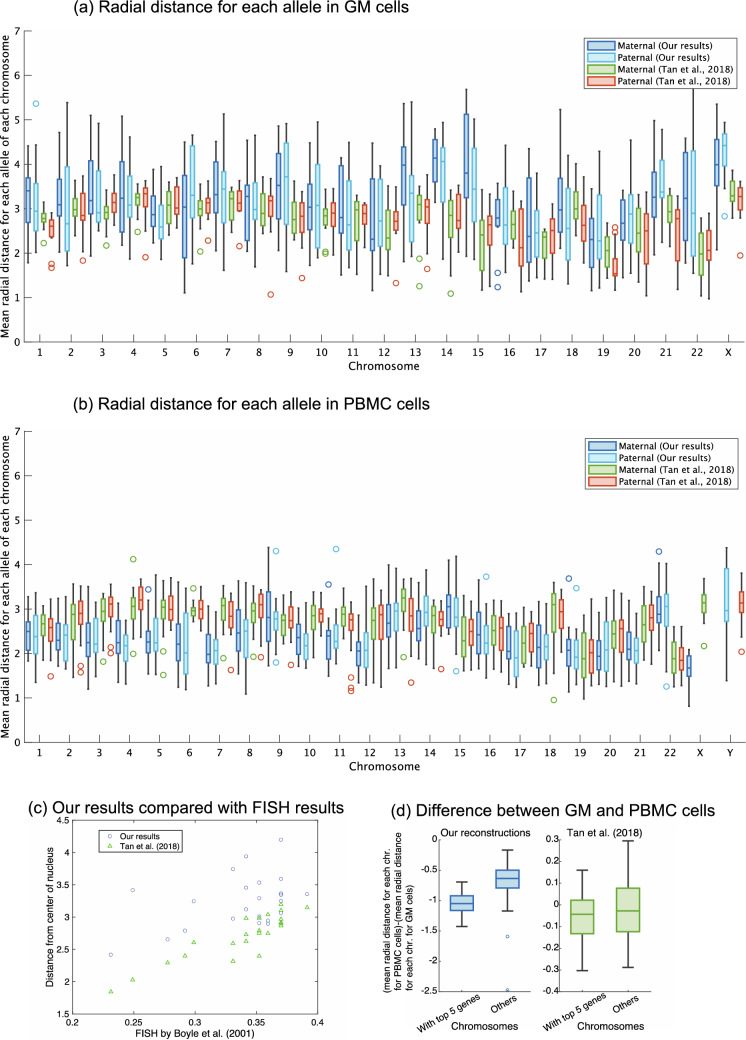
Comparisons of the reconstructed structures by radial distances for each allele at a 40-kb resolution. Panels (**a**) and (**b**) show boxplots of the radial distribution distributions for the corresponding alleles for GM cells and PBMC cells, respectively. For each chromosome, from left to right is our maternal allele, our paternal allele, the maternal allele from Ref.^13^, and the paternal allele from Ref.^13^, except for the sex chromosomes for PBMC cell in panel (**b**), where the left is our reconstructions and right is those of Ref.^13^. Here, GM cells 01 and 04 are excluded because they have some chromosomes without contact information. Panel (**c**) shows the scatterplot of the mean radial distance over all valid cells of our GM cell reconstructions versus the FISH results in Boyle et al*.* (2001). Here, we also plotted GM cell reconstructions by Tan et al. (2018) versus the FISH results. Panel (**d**) highlights the differences in the radial distances between PBMC cells and GM cells in the top five genes differentially expressed in PBMC cells^31^. According to Ref.^31^, the top five differentially expressed genes are located on chromosomes 7, 3, 15, 4, and 14.

The differences in Sect. 3.2 lead to the following qualitative differences. Our reconstructions reveal that one of the X chromosomes (possibly an inactive one) in female-derived GM cells is in the nuclear periphery, which is enriched with heterochromatin (Fig. 4a). On the other hand, the active X chromosome in male-derived PBMC cells is closer to the center of the nucleus, which has a higher abundance of euchromatin (Fig. 4b). This tendency is not observed in the reconstructions by Ref.^13^ (Fig. 4b). In addition, the radial distances in our reconstructions for GM cells correlate well with those obtained by the FISH data for lymphoblast nuclei^30^ (correlation coefficient: 0.4369; Fig. 4c). The reconstructions for GM cells by Tan et al.^13^ also shows high correlation (Fig. 4c; correlation coefficient: 0.8894). On the other hand, those for PBMC cells for our reconstructions are not correlated with the FISH data for lymphoblast nuclei (correlation coefficient: –0.0499). This may be due to the mismatch of cell types and the different shapes of chromosomes between GM cells and PBMC cells (the middle panel of Supplementary Fig. 6(d)). In our reconstructions, chromosomes with the top five over-expressed genes in PMBC cells tend to be closer to the center of the nucleus than in GM cells (Fig. 4d). Such tendencies are not observed in the reconstructions for PBMC cells by Ref.^13^ (Fig. 4d), while their reconstructions for male-derived PBMC cells have strong correlations of 0.9033 with the FISH data for lymphoblast nuclei^30^.

In our reconstructions, the maternal copies of the X chromosomes in the female-derived GM cells tend to be located closer to the center of the nuclei than the corresponding paternal ones (Fig. 4a; 10 out of 15 GM cells). This observation is consistent with the previous observation in GM cells^13^, the maternal X chromosome is actively transcribed, while they claimed in the same paper, the paternal X chromosomes likely resided at closer positions to the nuclei center than the maternal ones (Fig. 4a; 10 out of 15 GM cells). One of the two X homologous chromosomes in female-derived GM cells took a shape similar to that of the unique transcriptionally active X chromosome in male-derived PBMC cells. Choice of the active shaped X homologous chromosomes varies from cell to cell, which is consistent with the results by Tan et al.^13^ (Supplementary Fig. 7). All these results suggest that our new algorithm realizes more accurate reconstructions of the entire chromosome structures using a sparse Hi-C dataset from single diploid cells.

## Discussion

In summary, we propose a new method to analyze a sparse Hi-C dataset of a single diploid cell with a recurrence plot-based technique. Only phased pairs are used to reconstruct the chromosome structure. Then our reconstruction is refined using an analogy of the nonlinear time series prediction. Compared to the previous reconstruction, checking the reconstruction consistency with phased pairs, half-phased pairs, and unphased pairs improves the consistency. We also demonstrate that human chromosomes take fractal shapes and form chromosomal territories. In addition, our reconstructions provide consistent results that active chromosomes are located closer to the center of the nucleus, while inactive chromosomes are located in the nuclear periphery. We hope that the proposed method will be useful to reconstruct the chromosome structure more faithfully with a given single diploid cell Hi-C dataset.

The proposed method works even for sparse single diploid cell Hi-C data. There are two main reasons: (i) the local reconstruction distance is estimated by the ratio of the common neighbors to one of the two nearest neighbors. This ratio is robust and can be approximated even from sparse data as long as the SNVs occur randomly; (ii) the window size of the proposed analysis for the primary reconstruction (1-Mb) is sufficient to obtain a complete contact map without SNV frequency bias in the datasets used in this study.

About the reason (i), the local distances are estimated by the ratios of common neighbors for two points against neighbors for one of the two points (see Supplementary Text). Because such ratios can be satisfactorily approximated as long as the contacts are removed randomly, one can obtain the local distances robustly and the underlying metric space.

About the reason (ii), let us denote, by $q$, the probability that a contact is detected in Hi-C data where there is a contact in 40-kb solution. Then, in the refinement process, we increase the resolution by $W=25$. Then, the probability that we can have a contact in 1-Mb resolution can be written as $p=1-{(1-q)}^{{W}^{2}}$. If $q=0.01$ and $W=25$, $p\sim 0.998$. If there is no contact within the corresponding box of the contact map in 1-Mb resolution ($q=0$) and $W=25$, then, $p=0$. Thus, even if only 1% of all the contacts are available, we can obtain an almost perfect contact map in 1-Mb resolution. Due to these two reasons, we could reconstruct 3D chromosomal structures even from sparse data of phased pairs for single diploid cell Hi-C data (observe Supplementary Fig. 3).

We chose the resolution of 1-Mb for the primary reconstruction by the above reasons. The window size for the finer reconstruction (100-kb or 40-kb) was chosen depending upon the available computer memory and computation time limitations.: Currently, one need O(*N*^*2*^) memory space if the final resolution contains *N* points. Thus, overcoming this problem is one of our future research topics.

We also compared our method with the previously reported methods. The imputation-based method by Tan et al.^13^ assumed that (i) two alleles are spatially separated (Fig. 2e) and (ii) two alleles take different shapes (Supplementary Fig. 6(c)). On the other hand, we only assumed that two consecutive points of reconstructions on the same chromosomes are close to each other. The reconstructions for GM cells as well as PBMC cells by Tan et al.^13^ have the higher correlation at specific loci (especially loci used for the FISH analysis^30^) in lymphoblast cells than our reconstructions (Fig. 4c). However, our reconstructions are more consistent with the given Hi-C datasets of single human diploid cells or biological hallmarks of overall chromosome structures. For instance, our method has strength in (a) reconstruction consistency for phased pairs (Fig. 3); (b) visualization of the nucleolus compartment (Supplementary Fig. 4); (c) more likelihood radial distance for the maternal X chromosome in GM cells (Fig. 4a); (d) more likelihood radial distances for chromosomes with highly-expressed genes for PBMC cells (Fig. 4d). Although we do not know the reason for such differences, but some of them may be due to any assumptions or imputation processes in the method by Tan et al.^13^.

Microscopy-based methods such as multicolor FISH and oligonucleotide FISH will also enable us to precisely analyze the structure of specific chromosome regions^1^, particularly in combination with super-resolution microscopy. These methods are advantageous for obtaining statistical data on the spatial information of multiple cells and for revealing the spatial relationships of specific loci. On the other hand, single-cell Hi-C is advantageous for comprehensive analysis because it provides spatial coordinate information for the entire chromosome length. Our method is expected to further enhance the advantages of Hi-C analysis of various types of cells, because it can automatically infer unique chromosome structures from single-cell Hi-C data and distinguish homologous chromosome pairs. Combination of those methods is expected to accelerate the research on chromosomal structures and functions.

## Supplementary Information

Supplementary Information.

## Data Availability

All the necessary codes for reproducing this work can be found at https://doi.org/10.5281/zenodo.6562620. The results of our reconstructions can be found at https://doi.org/10.5061/dryad.3j9kd51hv.
