# Supplementary material for: Imputation-free reconstructions of three-dimensional chromosome architectures in human diploid single-cells using allele-specified contacts
Source: Sci Rep. 2022 Jul 11;12:11757. doi: 10.1038/s41598-022-15038-4 (PMC9273635; doi:10.1038/s41598-022-15038-4)
Supplement: Supplementary file 1 — Supplementary Information. [file 41598_2022_15038_MOESM1_ESM.pdf]

Supplementary Material

Supplementary Text

1. Mathematical details

Recurrence plots

Suppose that a series $\{x(i)|i = 1,2,\ldots,I\}$ is given with a defined threshold ε. In a Hi-C experiment, this threshold corresponds to the distance where allele pairs are detected as neighbors or a contact. Then a recurrence plot R (Eckmann *et al.*, 1987; Marwan *et al.*, 2007) is defined as

$$R(i,j) = \begin{cases} 1, & \text{if } d\big(x(i),x(j)\big) \leq \varepsilon, \\ 0, & \text{otherwise.} \end{cases}$$

If $R(i,j) = 1$, a point at (i,j) is plotted in the two-dimensional plane. If $R(i,j) = 0$, then a point at (i,j) is not plotted.

Reconstruction of the original three-dimensional chromosome structure from single diploid cell Hi-C data

A lot of information seems to be lost when converting a series into a recurrence plot. However, we can recover the rough shape of the original series from a recurrence plot (Thiel *et al.*, 2004; Hirata *et al.*, 2008). In particular, reconstruction using the method by (Hirata *et al.*, 2008) is supported by proofs (Hirata *et al.*, 2015; Khor and Small, 2016). This study also uses the method of (Hirata *et al.*, 2008) to infer the three-dimensional structure for chromosomes of a single diploid cell from its corresponding Hi-C dataset as follows. Suppose that for a cell line, we array the first maternal alleles of chromosomes from 1 to X followed by the second paternal alleles of chromosomes from 1 to X in females and from 1 to Y in males. We use this line as the axis for the series. Let a contact map for a single diploid cell be given as $R(i,j)$. Namely, if there is a contact between allele segments i and j at a 40-kb resolution and both contain single nucleotide variations, then set $R(i,j) = 1$. Otherwise, set $R(i,j) = 0$.

The following steps are used to reconstruct the three-dimensional structure of diploid chromosomes at a 1-Mb resolution and subsequently refine the three-dimensional structure at a 40-kb or 100-kb resolution.

Step (1): Declare that consecutive (C =) 25 or 10 segments from a contact pair on the same allele of the same chromosome are spatial neighbors when the chromosome structure is reconstructed at a 40-kb or 100-kb resolution, respectively: If $R(i,j) = 1$, allele segment i and allele segment $i + k$ are on the same allele of the same chromosome. Additionally, allele

segment j and allele segment $j+l$ are also on the same allele of the same chromosome. Then set $S(i+k, j+l)=1$ and $S(j+l, i+k)=1$ for each $k, l=-C, -C+1, \ldots, \text{ or } C$.

Step (2): Let $C_2 = \lceil C/2 \rceil$. If $S(C_2+Ci, C_2+Cj)=1$, reconstruct the local distance $d(C_2+C/2i, C_2+Cj)$ between allele segments C_2+Ci and C_2+Cj in the 1-Mb resolution by

$$d(C_2+Ci, C_2+Cj)=\frac{a(C_2+Ci, C_2+Cj)}{b(C_2+Ci, C_2+Cj)} \quad (1)$$

where

$$a(C_2+Ci, C_2+Cj) = \sum_m S(C_2+Ci, C_2+Cm)+\sum_m S(C_2+Cj, C_2+Cm) - 2\sum_m S(C_2+Ci, C_2+Cm)S(C_2+Cj, C_2+Cm)$$

and

$$b(C_2+Ci, C_2+Cj) = \sum_m S(C_2+Ci, C_2+Cm)+\sum_m S(C_2+Cj, C_2+Cm) - \sum_m S(C_2+Ci, C_2+Cm)S(C_2+Cj, C_2+Cm).$$

Namely, variable a counts the number of neighbors not shared between points C_2+Ci and C_2+Cj in the 1-Mb resolution, while variable b counts the number of neighbors contained in either point of C_2+Ci or C_2+Cj in the 1-Mb resolution. Then construct a graph with edges where the local distances are attached in the 1-Mb resolution. If all the nodes are connected in this graph, move to the next step. Otherwise, the three-dimensional structure of the chromosomes cannot be reconstructed.

Step (3): Find the shortest distance between every pair of nodes on this graph and estimate the global geodesic distance $D(C_2+Ci, C_2+Cj)$ in the 1-Mb resolution for every pair (i, j) of nodes. In this step, various approaches are possible. One approach is the Dijkstra method (Dijkstra, 1959). Another is the Johnson method (Johnson, 1977).

Step (4): Find a set of point configurations such that the above global geodesic distances are

preserved by multidimensional scaling (Gower, 1966). If the top three eigenvalue components are selected, our reconstruction is obtained for the three-dimensional structure for a single diploid cell at the 1-Mb resolution. This reconstruction is described by the three-dimensional series as $\{x(C_2 + Cj)\}$.

Step (5): As an additional step newly introduced here, refine the reconstruction by borrowing an idea from a time series prediction. For each segment i, find, for each j, the following local distance $\Delta(i, C_2 + Cj)$ as

$$\Delta(i, C_2 + Cj) = \frac{A(i,C_2+Cj)}{B(i,C_2+Cj)} \qquad (2)$$

where

$$A(i, C_2 + Cj) = \sum_m S(i,m) + \sum_m S(C_2 + Cj, m) - 2\sum_m S(i,m)S(C_2 + Cj, m)$$

and

$$B(i, C_2 + Cj) = \sum_m S(i,m) + \sum_m S(C_2 + Cj, m) - \sum_m S(i,m)S(C_2 + Cj, m).$$

Then find the four nearest neighbors. Let $J(i)$ be the set of indices for the four nearest neighbors of i. Then a weight $w(i, C_2 + Cj)$ for each $j \in J(i)$ is given as

$$w(i, C_2 + Cj) = \frac{\exp\left(-\Delta(i, C_2 + Cj)\right)}{\sum_{l \in J(i)} \exp(-\Delta(i, C_2 + Cl))}.$$

Lastly, a reconstruction is obtained for segment i at a 40-kb or 100-kb resolution as

$$y(i) = \sum_{j \in J(i)} w(i, C_2 + Cj)x(C_2 + Cj).$$

2. Derivation for the detection limit length of Hi-C data in the proposed method

In the proposed method, the detection limit length corresponds to threshold ε, which is a constant provided from the experimental data. If the distance between two points is at the detection limit length, the distance coincides with ε. Equation (1) can be changed in the following way:

$$d(C_2 + Ci, C_2 + Cj) = 1 - \frac{\sum_m S(C_2 + Ci, C_2 + Cm)S(C_2 + Cj, C_2 + Cm)}{b(C_2 + Ci, C_2 + Cj)}.$$

Here in the second term of the right-hand side, the numerator can be interpreted as the number of points where points i and j simultaneously have the contacts with points $C_2 + Cm$, while the

denominator can be regarded as the number of points where either i or j has a contact with points $C_2 + Cm$,. Thus, if we approximate the ratio of points defined by Eq. (1) with the ratio of the corresponding volumes in three-dimensional space (Supplementary Figure 8), the local distance $\bar{d}$ of Eq. (1) at the detection limit can be written using two overlapping spheres as

$$\bar{d} \sim \frac{2\int_{-\varepsilon}^{\varepsilon} \pi(\varepsilon^2 - x^2)dx - 2 \times 2\int_{\frac{1}{2}\varepsilon}^{\varepsilon} \pi(\varepsilon^2 - x^2)dx}{2\int_{-\varepsilon}^{\varepsilon} \pi(\varepsilon^2 - x^2)dx - 2\int_{\frac{1}{2}\varepsilon}^{\varepsilon} \pi(\varepsilon^2 - x^2)dx} = \frac{\int_{-\varepsilon}^{\varepsilon} (\varepsilon^2 - x^2)dx - 2\int_{\frac{1}{2}\varepsilon}^{\varepsilon} (\varepsilon^2 - x^2)dx}{\int_{-\varepsilon}^{\varepsilon} (\varepsilon^2 - x^2)dx - \int_{\frac{1}{2}\varepsilon}^{\varepsilon} (\varepsilon^2 - x^2)dx}.$$

We have

$$\int_{-\varepsilon}^{\varepsilon} (\varepsilon^2 - x^2)dx = \left[\varepsilon^2 x - \frac{1}{3}x^3\right]_{-\varepsilon}^{\varepsilon} = \frac{4}{3}\varepsilon^3,$$

and

$$\int_{\frac{1}{2}\varepsilon}^{\varepsilon} (\varepsilon^2 - x^2)dx = \left[\varepsilon^2 x - \frac{1}{3}x^3\right]_{\frac{1}{2}\varepsilon}^{\varepsilon} = \frac{2}{3}\varepsilon^3 - \frac{11}{24}\varepsilon^3 = \frac{5}{24}\varepsilon^3.$$

Thus, we have

$$\bar{d} \sim \frac{\frac{4}{3} - 2\frac{5}{24}}{\frac{4}{3} - \frac{5}{24}} = \frac{32 - 10}{32 - 5} = \frac{22}{27}.$$

Therefore, our proposed method uses $\frac{22}{27}$ for the length of the detection limit of Hi-C data in the calculation. A similar story holds for Eq. (2) as well.

3. Time complexity of the proposed algorithm

Let M be the number of points for a coarse reconstruction, and N be the number of points for a finer reconstruction. Let E be the mean number of contacts for each segment in the coarse reconstruction. Here, assume that $E \sim cN$. Step (1) prepares a matrix S in $O(N^2)$. Step (2) requires $O(M^3)$ calculations to construct a graph for a coarse resolution. Step (3) finds the all-to-all shortest distances using Johnson's method in $O(M^2 \log M + EM) \sim O(M^2 \log M + cMN)$ (Cormen, Thomas H. ; Leiserson, Charles E. : Rivest, Ronald L. ; Stein, 2009). Step (4) identifies the coarse configuration by multidimensional scaling in $O(M^3)$ (Gower, 1966). Step (5) refines the resolution in $O(MN^2)$. Overall, the algorithm runs in $O(M^3 + MN^2)$, which is $O(N^2)$ if M is regarded as a fixed constant. Thus, our approach partially overcomes the problem of $O(N^3)$ time complexity, which impacts most existing methods (MacKay and Kusalik, 2020). For example, obtaining the results for Cell GM 9 in (Tan *et al.*, 2018) requires 33.95 minutes using a computer with a CPU (2.6GHz 6 Core Intel Core i7) and 64 GB memory via MATLAB.

4. Validation using protein/polymer models

First, we tested the proposed algorithm before the refinement with protein data, which are

polymers of amino acids. We used the data of 14 proteins, which are given by the distances between every pair of points. The test protein set was constructed from the CASP13 targets from https://www.predictioncenter.org/download_area/CASP13/targets/, with the official domain definitions (Kryshtafovych *et al.*, 2019; Kinch *et al.*, 2019). We chose all proteins classified as "Free Modeling (FM)" with structures deposited at the Worldwide Protein Data Bank as of March 2020. The length of the resulting proteins ranged between 72–374 amino acids. Supplementary Table 2 lists the proteins used in this study. Two residues are in contact if the distance between two Cα atoms of these residues is below the threshold. We varied the threshold from 6 Å to 15 Å in 1 Å increments to generate an original contact map for each case. After the reconstruction, we matched the scale for each reconstruction so that the mean distance between points i and $i+1$ for each i was 3.8 Å (Supplementary Fig. 2). In these examples, our reconstructions tend to be more accurate from the viewpoint of the 3D correlation coefficient (Hirata *et al.*, 2016) than that of (Lesne *et al.*, 2014) when the local distance is set to 1 without using the Jaccard coefficient. See Eq. (1) in the Supplementary Material for more details.

5. Evaluating the ratio of intra-chromosomal contacts

We evaluated the distances between all pairs of representative points at a 100-kb resolution. If the distance is less than 22/27, which is the Hi-C detection limit, then the corresponding pair is recorded as neighboring. Otherwise, this pair is not neighboring. For each allele on each chromosome, we found the ratio of intra-contacts among all the contacts for each cell.

6. Analysis for the space of nucleolus

We examined whether there is a vacant space in the center of our reconstruction using the following method. First, find the box containing all the points of our reconstruction for a cell using the above approach. Second, divide the box into 20x20x20 segments. Finally, check that there is at least one empty box within the 6x6x6 boxes at the center.

7. Evaluating the ability of our reconstructions to separate neighboring points from other points far apart

We investigated this by making 2x2 tables and comparing whether the distance is within the Hi-C detection limit or whether the corresponding pair is a phased contact. Below, the results are summarized for GM cells only because for PBMC cells, about 80% of the phased contacts are those of the maternal Chr X.

Supplementary Table 3 shows an example for GM cell 2 with the proposed method with a 1-

Mb band width (25 points). Supplementary Table 4 presents the corresponding overall results for GM cells. These tables clearly demonstrate that the proposed method successfully classifies the phased contacts as spatial neighbors, while classifying most other points as those far away without such phased contacts. The odds ratios for Supplementary Tables 3 and 4 are 2437.9 and 1003.2, respectively. Thus, the specificity for the proposed method is high.

Supplementary Table 5 shows an example for GM cell 2 by Tan *et al.* (2018). Supplementary Table 6 presents the corresponding overall results. Although the method of Tan *et al.* (2018) can successfully classify contact points, its specificity is inferior to the proposed method because the odds ratios for Supplementary Tables 5 and 6 are 142.7 and 157.0, respectively.

8. Effects of the band width in our reconstruction

We evaluated the effects of the band width using an approach similar to Supplementary Text Section 7.

Supplementary Tables 4, 7, 8, and 9 correspond to cases with band widths of 25 (results in the main text), 50, 125, and 250, respectively. The odds ratios are 1003.2, 450.7, 251.2, and 181.0, respectively. Therefore, reconstructions with a band width of 25 have the highest specificity among the tested thresholds.

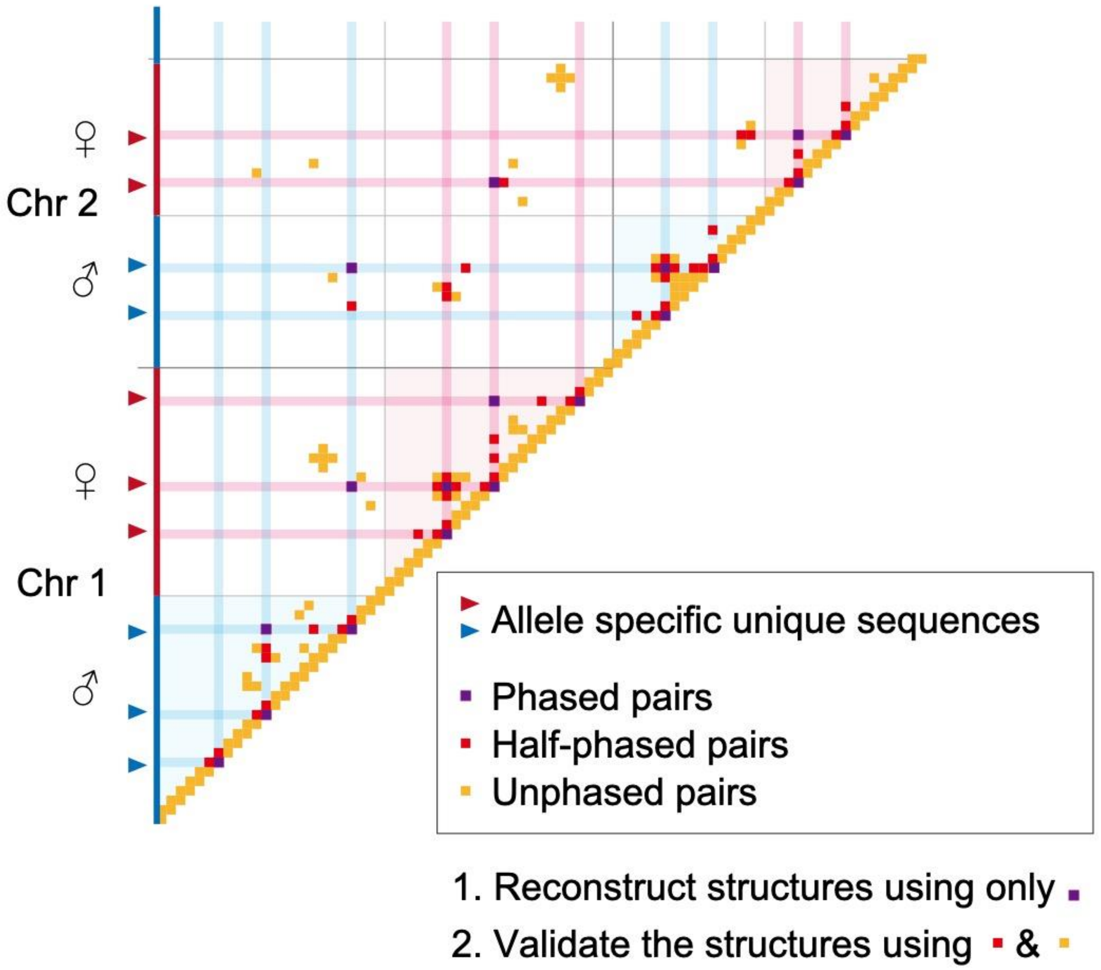

Supplementary Figure 1: Graphic summary for the proposed method for reconstructing three-dimensional chromosomal structures from single diploid cell Hi-C data.

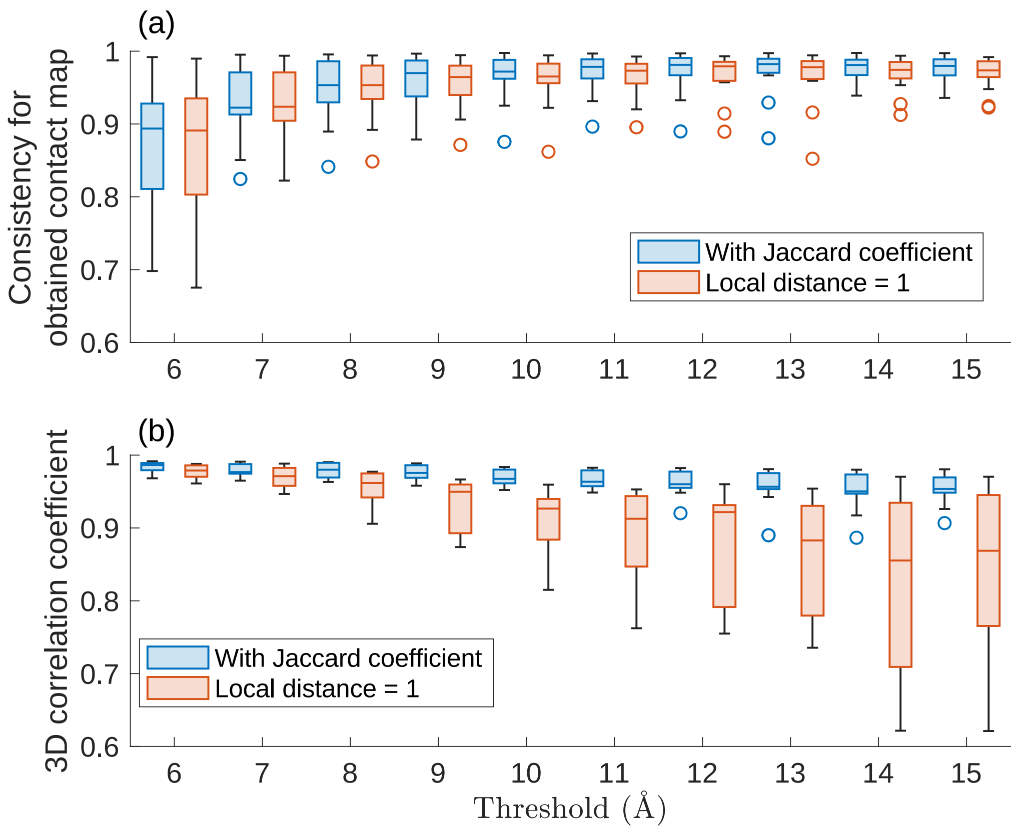

Supplementary Figure 2: Test for the proposed method using 14 different three-dimensional (3D) protein structures, depending on the threshold size. We test our procedure up to the fourth step prior to refinement. All data points are used for the reconstructions. Panel (a) shows how consistently a contact map is reproduced after reconstructing each 3D structure. Panel (b) shows how large the 3D correlation coefficient (Hirata *et al.*, 2016) is between the original 3D structures and their reconstructions. The 3D correlation coefficient provides the correlation coefficient between the distance matrix among the points between two 3D structures. The more similar two structures are, the closer the value is to one. For the mathematical validity of the 3D correlation coefficient, please see the Supplementary Material of (Hirata *et al.*, 2016). We compare the proposed method ("With Jaccard coefficient") with that of (Lesne *et al.*, 2014) ("Local distance=1"). We simplified the comparison by defining every local distance as 1 rather than using Eq. (1) based on the Jaccard coefficient. In these panels, the box plots show the distribution: the central boxes show the first and third quarters of each distribution. The middle line shows the median of the distribution. Minimum and maximum within the 1.5 interquartile range from the central box are shown by the "whiskers." Circles denote outliers.

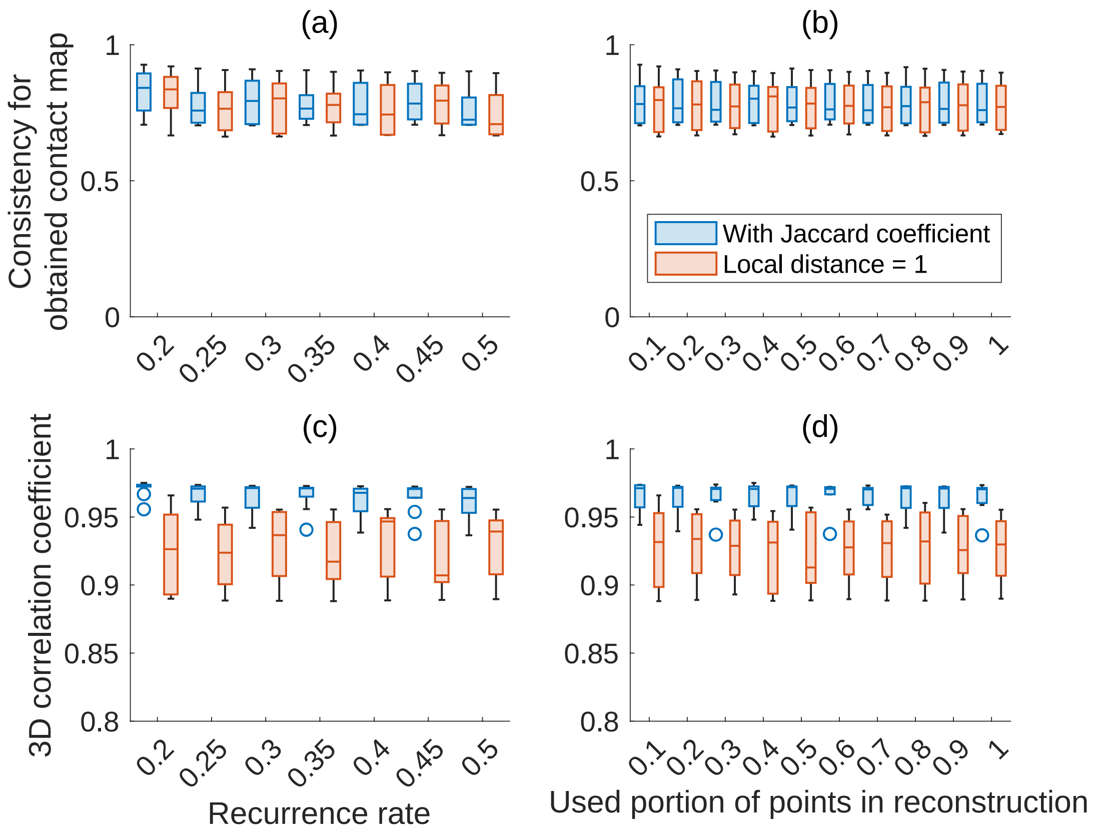

Supplementary Figure 3: Test of the proposed method using a polymer model simulation at a 1-Mb resolution depending on the threshold level (recurrence rate) and the portion of contacts used for our reconstructions. Panels (a) and (b) evaluate the consistency between the original contact map and the contact map obtained after reconstructing the three-dimensional structure. Panels (c) and (d) evaluate the 3D correlation coefficient. Panels (a) and (c) summarize our results according to the recurrence rate or how we chose the threshold. Panels (b) and (d) summarize our results according to the portion of points used in our reconstruction. Namely, if the portion is 0.1, 90% of points in the contact map are discarded. In each panel, “With Jaccard coefficient” corresponds to the results by the proposed approach, while “Local distance=1” corresponds to the results by the approach of (Lesne *et al.*, 2014). Results can be interpreted similar to those in Supplementary Figure 2.

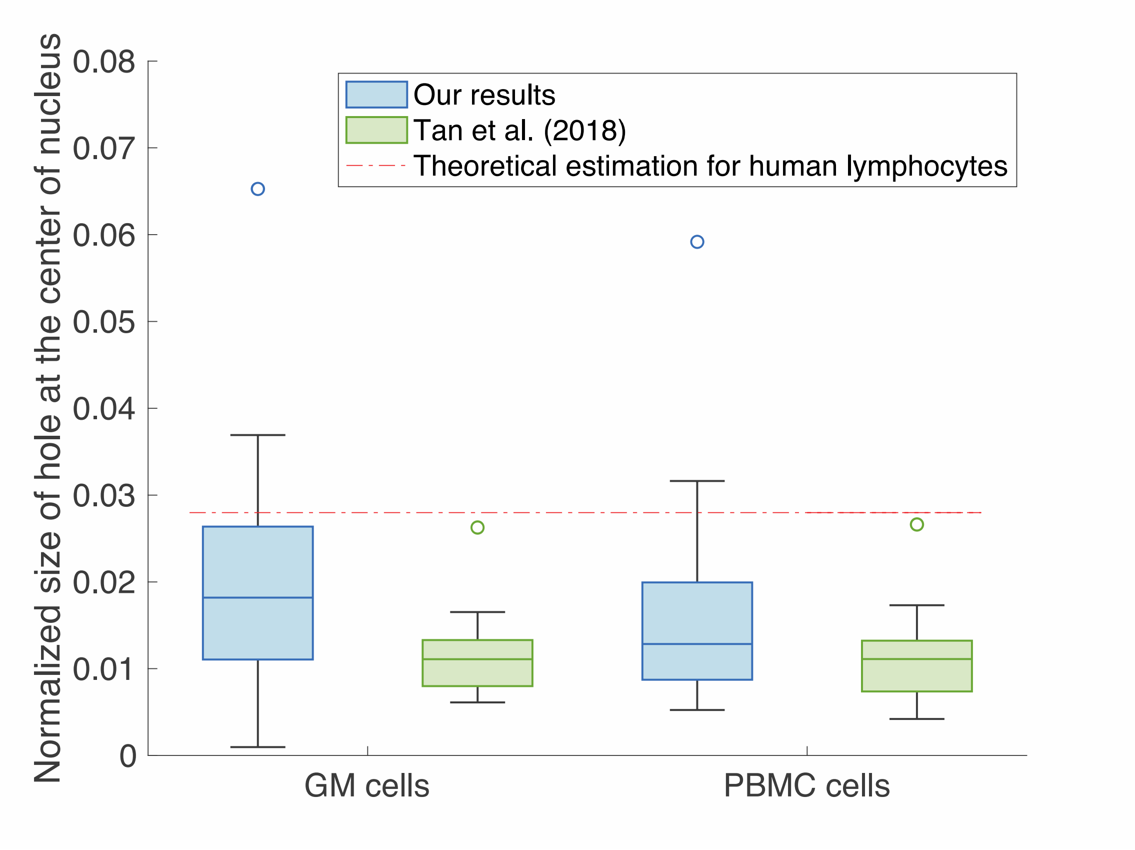

Supplementary Figure 4: Radius of the hole at the center of the nucleus normalized at the maximum radial distance of the reconstructed points. Red-dashed line is the value obtained from the nucleolar area (Berger, 2008) and the nucleus volume (Loiko *et al.*, 2006) for human lymphocytes.

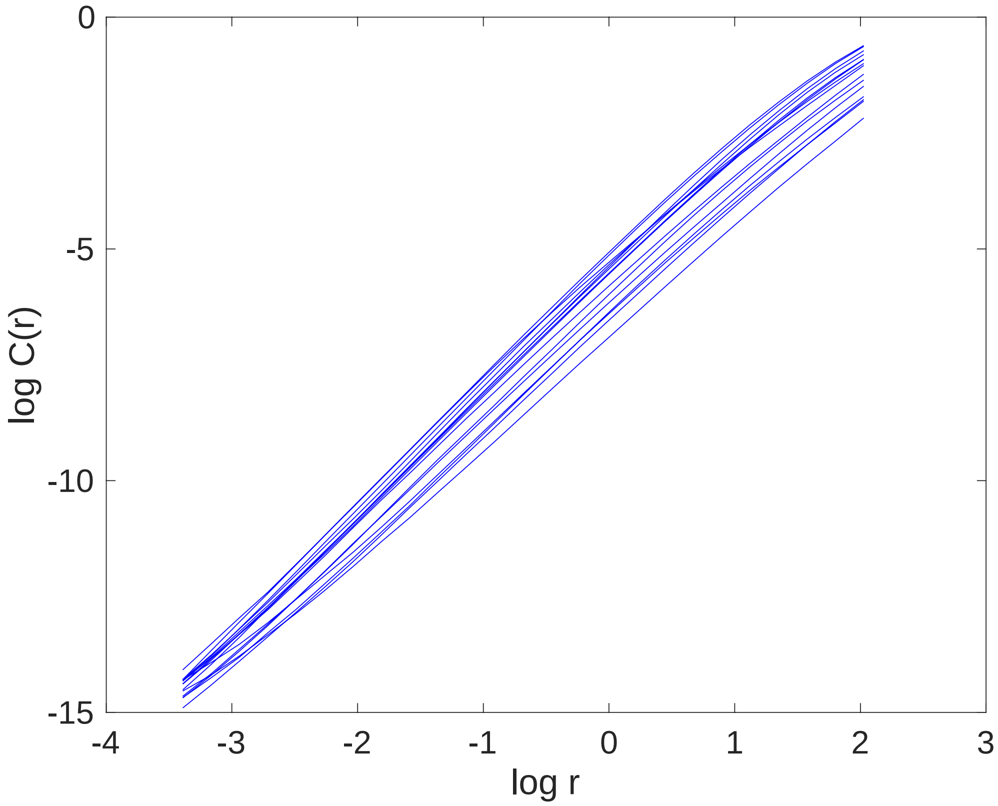

Supplementary Figure 5: Intermediate figure to obtain the correlation coefficients for 15 GM cells whose reconstructions could be completed by the proposed method. Note that we can take the scaling region in the presented range of log r, which corresponds to the logarithm of the spatial distance.

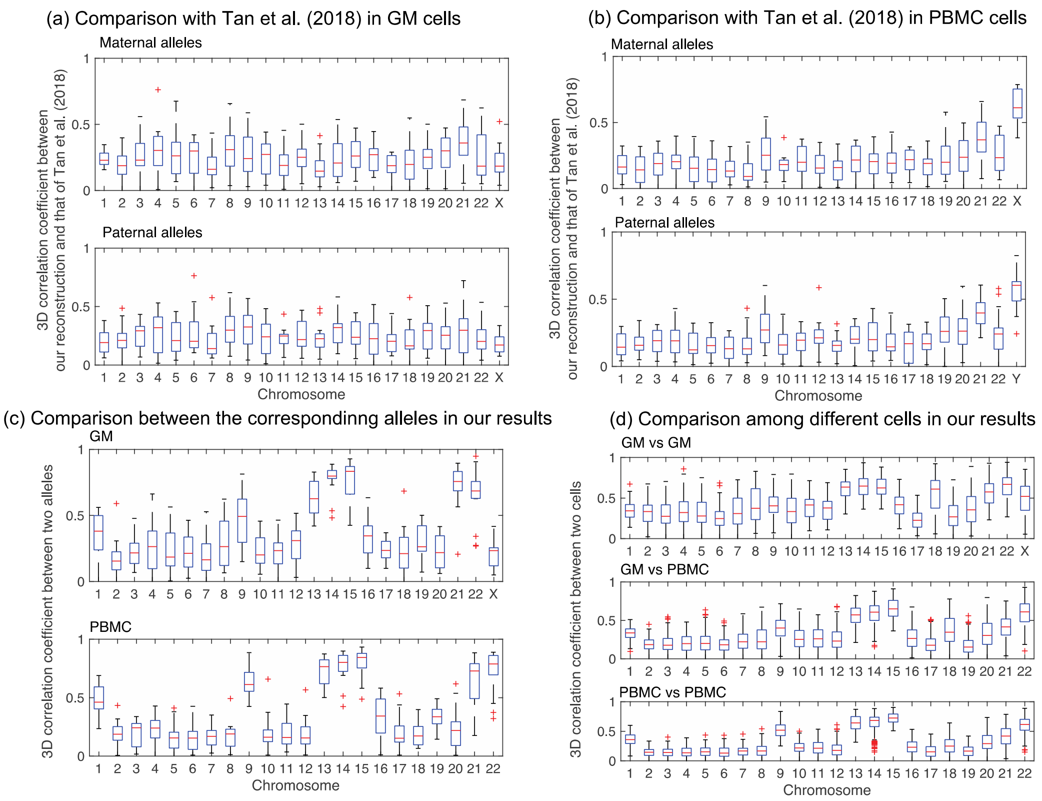

Supplementary Figure 6: Comparisons of our reconstructions using boxplots of the 3D correlation coefficients. Panels (a) and (b) compare our reconstructions for GM cells and PBMC cells, respectively, with those of Tan *et al.* (2018). Panel (c) compares two alleles of the same chromosome within the same cell. Panel (d) compares the chromosomes in one cell with those in another. Larger values closer to 1 indicate that the corresponding 3D structures are more similar.

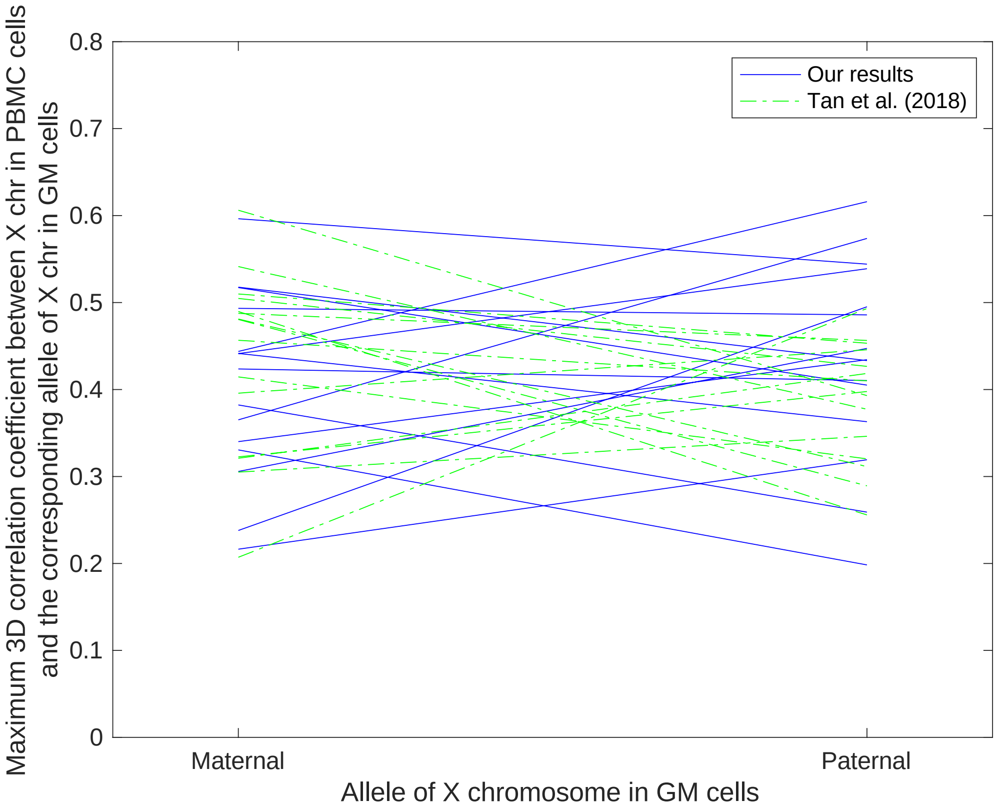

Supplementary Figure 7: Which allele of X chromosome in female-derived GM cells looks similar to the X chromosome in male-derived PBMC cells. Here we compared the maximum for 3D correlation coefficient between an allele of X chromosome in GM cells and the X chromosome in PMBC cells over all the PBMC cells. Each line corresponds to an GM cell.

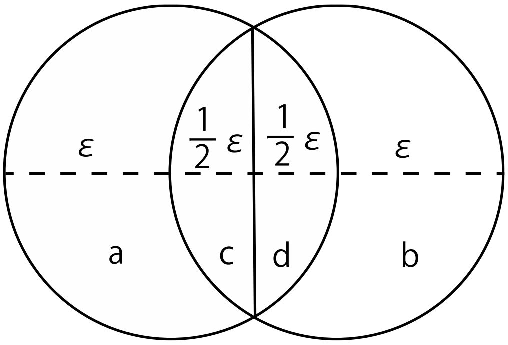

Supplementary Figure 8: Schematic figure for approximating Eq. (1) with the volumes of two spheres. The local distance $\bar{d}$ can be estimated by (the sum of the volumes for regions a & b)/(the sum of the volumes for regions a, b, c & d) according to Eq. (1).

Supplementary Table 1: Number of Hi-C contact pairs for each analyzed cell and the proportion of phased, half phased, and unphased pairs.

Cell	Total pairs	Phased pairs	Half-phased pairs	Unphased pairs
GM01	657377 (100%)	5472 (0.83%)	102559 (15.6%)	549346 (83.6%)
GM02	662037 (100%)	5310 (0.80%)	101862 (15.4%)	554865 (83.8%)
GM03	802576 (100%)	7347 (0.92%)	131518 (16.4%)	663711 (82.7%)
GM04	910625 (100%)	9696 (1.06%)	157957 (17.4%)	742972 (81.6%)
GM05	748508 (100%)	7200 (0.96%)	128097 (17.1%)	613211 (81.9%)
GM06	753158 (100%)	6822 (0.91%)	122533 (16.3%)	623803 (82.8%)
GM07	899189 (100%)	9209 (1.02%)	153134 (17.0%)	736846 (82.0%)
GM09	943049 (100%)	9000 (0.95%)	155132 (16.5%)	778917 (82.6%)
GM11	1126475 (100%)	10362 (0.92%)	183123 (16.3%)	932990 (82.8%)
GM12	1029942 (100%)	10242 (0.99%)	167912 (16.3%)	851788 (82.7%)
GM13	1065539 (100%)	11494 (1.08%)	177571 (16.7%)	876474 (82.3%)
GM14	1026524 (100%)	12653 (1.23%)	170532 (16.6%)	843339 (82.1%)
GM15	1055182 (100%)	9933 (0.94%)	168767 (16.0%)	876482 (83.1%)
GM16	992536 (100%)	9342 (0.94%)	165870 (16.7%)	817324 (82.4%)
GM17	1026364 (100%)	9586 (0.93%)	163527 (15.9%)	853251 (83.1%)
PBMC01	749242 (100%)	7260 (0.97%)	122030 (16.3%)	619952 (82.7%)
PBMC02	925232 (100%)	8282 (0.90%)	149291 (16.1%)	767659 (83.0%)
PBMC03	759194 (100%)	7020 (0.92%)	124719 (16.4%)	627455 (82.7%)
PBMC04	791556 (100%)	7435 (0.94%)	129178 (16.3%)	654943 (82.7%)
PBMC05	685878 (100%)	6724 (0.98%)	115639 (16.9%)	563515 (82.2%)
PBMC06	778976 (100%)	7492 (0.96%)	127247 (16.3%)	644237 (82.7%)
PBMC07	823505 (100%)	7978 (0.97%)	133140 (16.2%)	682387 (82.9%)
PBMC08	742170 (100%)	6837 (0.92%)	120360 (16.2%)	614973 (92.9%)
PBMC09	910034 (100%)	8362 (0.92%)	150520 (16.5%)	751152 (82.5%)
PBMC10	733695 (100%)	6636 (0.90%)	117747 (16.1%)	609312 (83.1%)
PBMC11	683784 (100%)	6110 (0.89%)	108829 (15.9%)	568845 (83.2%)
PBMC12	680583 (100%)	6040 (0.89%)	108142 (15.9%)	566401 (83.2%)
PBMC13	703694 (100%)	6154 (0.87%)	112185 (15.9%)	585355 (83.2%)
PBMC14	995614 (100%)	8173 (0.82%)	155196 (15.6%)	832355 (83.2%)
PBMC15	713076 (100%)	6413 (0.90%)	114183 (16.0%)	582480 (83.6%)
PBMC16	596832 (100%)	5800 (0.97%)	99234 (16.6%)	491798 (82.4%)
PBMC17	796327 (100%)	7147 (0.90%)	128622 (16.2%)	660558 (83.0%)

PBMC18	909649 (100%)	7860 (0.86%)	144876 (15.9%)	756913 (83.2%)

Supplementary Table 2: List of the test protein sets used in this study.

CASP target ID	PDB ID_chain ID
T0950	6ek4_A
T0953s1	6f45_B
T0953s2	6f45_D
T0960	6cl5_A
T0968s1	6cp9_A
T0969	6cci_A
T0970	6g57_A
T0980s1	6gnx_C
T0986s2	6d7y_B
T0990	6n9y_A
T1000	6u7l_A
T1021s2	6rap_C
T1021s3	6rap_E
T022s1	6rbk_A

Supplementary Table 3: Classification results for the proposed method for GM cell 2 with a band width of 25. These numbers show the pairs of points for the corresponding categories.

	Phased contacts	Without phased contacts	Total
Spatial neighbors	3949	7211972	72123121
Points far away	257	11442135498	11442135755
Total	4206	11449347470	11449351676

Supplementary Table 4: Overall classification results for the proposed method for GM cells.

	Phased contacts	Without phased contacts	Total
Spatial neighbors	93337	2048166108	2048259445
Points far away	7379	162433439920	162433447299
Total	100716	164481606028	164481706744

Supplementary Table 5: Classification results from Tan *et al.* (2018) for GM cell 2.

	Phased contacts	Without phased contacts	Total
Spatial neighbors	2491	331250683	331253174
Points far away	2007	38077993360	38077995367
Total	4498	38409244043	38409248541

Supplementary Table 6: Overall classification results from Tan *et al.* (2018) for GM cells.

	Phased contacts	Without phased contacts	Total
Spatial neighbors	67619	5532973178	5533040797
Points far away	42179	541815041137	541815083316
Total	109798	547348014315	547348124113

Supplementary Table 7: Overall classification results for the proposed method for GM cells with a band width of 50. These numbers show the pairs of points for the corresponding categories.

	Phased contacts	Without phased contacts	Total
Spatial neighbors	93553	4631932986	4632026539
Points far away	7163	159849673042	159848680205
Total	100716	164481606028	164481706744

Supplementary Table 8: Similar results as Supplementary Table 7 except that the band width is set to 125.

	Phased contacts	Without phased contacts	Total
Spatial neighbors	94855	9954715063	9954809918
Points far away	5861	154526890965	154526896826
Total	100716	164481606028	164481706744

Supplementary Table 9: Similar results as Supplementary Table 7 except that the band width is set to 250.

	Phased contacts	Without phased contacts	Total
Spatial neighbors	95803	15993230306	15993326109
Points far away	4913	148488375722	148488380635
Total	100716	164481606028	164481706744

References

Berger,J. (2008) Nucleolar size in lymphocytes and haemocytes of different species. *Eur. J. Histochem.*, **52**, 149–152.

Cormen, Thomas H. ; Leiserson, Charles E. : Rivest, Ronald L. ; Stein,C. (2009) Introduction to Algorithm Third Edition Third edit. Massachusetts Institute of Technology, Cambridge, MA.

Dijkstra,E.W. (1959) A note on two problems in connexion with graphs. *Numer. Math.*, **1**, 269–271.

Eckmann,J.P. *et al.* (1987) Recurrence plots of dynamical systems. *Epl*, **4**, 973–977.

Gower,J.C. (1966) Some Distance Properties of Latent Root and Vector Methods Used in Multivariate Analysis. *Biometrika*, **53**, 325.

Hirata,Y. *et al.* (2015) Faithfulness of Recurrence Plots: A Mathematical Proof. *Int. J. Bifurc. Chaos*, **25**, 1550168.

Hirata,Y. *et al.* (2008) Reproduction of distance matrices and original time series from recurrence plots and their applications. *Eur. Phys. J. Spec. Top.*, **164**, 13–22.

Hirata,Y. *et al.* (2016) Three-dimensional reconstruction of single-cell chromosome structure using recurrence plots. *Sci. Rep.*, **6**.

Johnson,D.B. (1977) Efficient Algorithms for Shortest Paths in Sparse Networks. *J. ACM*, **24**, 1–13.

Khor,A. and Small,M. (2016) Examining k-nearest neighbour networks: Superfamily phenomena and inversion. *Chaos*, **26**, 043101.

Kinch,L.N. *et al.* (2019) CASP13 target classification into tertiary structure prediction categories. *Proteins Struct. Funct. Bioinforma.*, **87**, 1021–1036.

Kryshtafovych,A. *et al.* (2019) Critical assessment of methods of protein structure prediction (CASP)—Round XIII. *Proteins Struct. Funct. Bioinforma.*, **87**, 1011–1020.

Lesne,A. *et al.* (2014) 3D genome reconstruction from chromosomal contacts. *Nat. Methods*, **11**, 1141–1143.

Loiko,V.A. *et al.* (2006) Morphometric model of lymphocyte as applied to scanning flow cytometry. *J. Quant. Spectrosc. Radiat. Transf.*, **102**, 73–84.

MacKay,K. and Kusalik,A. (2020) Computational methods for predicting 3D genomic organization from high-resolution chromosome conformation capture data. *Brief. Funct. Genomics*, **19**, 292–308.

Marwan,N. *et al.* (2007) Recurrence plots for the analysis of complex systems. *Phys. Rep.*, **438**, 237–329.

Tan,L. *et al.* (2018) Three-dimensional genome structures of single diploid human cells. *Science (80-.).*, **361**, 924–928.

Thiel,M. *et al.* (2004) How much information is contained in a recurrence plot? *Phys. Lett. Sect. A Gen. At. Solid State Phys.*, **330**, 343–349.
